# Neglected problem: Influence of school bag on lumbar segment in children

**DOI:** 10.3389/fped.2022.1045666

**Published:** 2022-11-15

**Authors:** Milan Bajin, Milan Kojić, Romana Romanov, Zlatko Ahmetović

**Affiliations:** ^1^ Faculty of Sport and Psychology, Educons University in Novi Sad, Novi Sad, Serbia; ^2^ Faculty of Sports and Physical Education, University of Belgrade, Belgrade, Serbia

**Keywords:** photogrammetry, sagittal plane, lumbar angle, schoolchildren, standing posture, gait, backpack weight

## Abstract

**Background and Objectives:**

School bag (SB) load causes significant changes in the height and symmetry of the intervertebral discs at each level of the spine from T12-L1 to L5-S1. This study aims to determine the change in the size of the lumbar segment angle at a particularly critical point L3-L4 of the spine in relation to the load of the average weight of SB in healthy male children (students) at standing and after 2-minute gait.

**Methods:**

47 boys, aged 12.2 ± 0.92 years, underwent photogrammetric measurements in the sagittal plane in statics and dynamics, walking on a laboratory treadmill. Measurements were repeated with the weight of SB with a constant load of 6,251 kg, which represents 13.78% of the average body weight of our sample. The lumbar angle (LA) connecting the point of the big toe, the lumbar point L3-L4 and the processus spinosus C7 was measured. In gait, LA was measured in the phases of the middle support and the initial contact of the heel.

**Results:**

T-test of paired samples was used to estimate the change in LA at standing from 4.953° and walking phases from 6.295° to 7.332° in relation to the unloaded state, and the value of the effect size (ES) indicates that the impact of SB load is significant.

**Conclusions:**

Cumulatively, microtraumas caused by SB load significantly affect the increase in intervertebral pressure at the L3-L4 point, which is susceptible to degenerative processes and which can be the cause of lumbar syndrome (LS). Preventive measures are needed in order to lighten SB in this population and introduce up to 10% of students' body weight into the safe zone.

## Introduction

Researches, that have dealt with risk factors that affect the degenerative processes of the spine, suggest that this issue requires a complex analysis of their occurrence. In addition to genetic preconditions (1), anthropometric parameters (2) and microtrauma are the most common exogenous factors that can overload the spine during growth and development and thus have an impact on the etiology of degenerative processes (3–7). Exogenous factors, especially in the sensitive period of growth and development, can have a negative impact on posture (8, 9). One of the most frequently mentioned factors that intrigues the scientific public and worries parents is the load conditioned by the weight of the school bag - school backpack (SB) (10–16). A number of studies have identified that students most often carry SB on both shoulders (83%) (17), and that SB weight ranged from 10%–22% of the total body weight of students (18–24). The load caused by SB leads to an increase in axial pressure on the spinal column and causes changes in the height and symmetry of the intervertebral discs of the T12-S1 level, but also changes in the angular values of the intervertebral relations (25). The size, frequency and duration of the load conditioned by SB, additionally increases the risk of creating postural changes in students during the period of rapid growth and development (26, 27).

Previous researches, that have dealt with the problem of SB load on the spinal column and the reaction in the form of changing the angle for the torso segment, analyzed different angles. They have dealt with the angles formed between the points trochanterion (femoris) and acromion and in relation to the reference horizontal (18, 19, 21, 28, 29), i.e., vertical line that intersects the great trochanter of the femur (30–32). A group of German authors examined the angle of inclination of the torso in relation to the horizontal line, where the torso is defined as a vertical line formed by the mean values of anatomical points on its upper (both acromion, jugular fossa and processus spinosus C7) and lower segment (upper sacrum, left and right iliac bone - anterior spina iliaca superior) (33). The angle of inclination of the torso that forms the anatomical points on the big toe, the intervertebral space between L3-L4 and the processus spinosus C7, was the subject of a study that considered the change in angle under different loads due to carrying different types of women's bags (female subjects, aged 19 to 37) (34). In accordance with all the above, the angle of the lumbar segment that defines the anatomical points of the big toe, the intervertebral space L3-L4 and the processus spinosus C7, can be studied by considering additional load in the form of SB and in the student population. The reasons are primarily related to the understanding of anatomical-physiological structure, and forces influencing the lumbar spine segment located on the disc between L3-L4, which also forms the peak of the lordotic curve and is the riskiest place for degenerative changes (35, 36). Intervertebral pressure in resting phases such as standing, sitting without support, as well as all types of lying, is highest in the L3-L4 region (37). Anatomically, L4 has the smallest prosessus transverus and relatively long iliotransversal ligaments that provide less support and stability (38). The multifidus muscle maintains lumbar lordosis by acting as a tendon that helps transmit certain axial compression forces on the disc to the anterior longitudinal ligament by shifting from the compression to the tensile load and further protects the discs by preventing unexpected movements such as twisting and bending (39). Regardless of the more significant role which the paraspinal muscles have in the protection of the L3-L4 segment, the greatest correlation of multifidus muscle atrophy is in that region, which additionally makes this point susceptible to degenerative changes (36).

The large weight of SB on the student's back changes the center of gravity of the body and conditions the adjustment to the posterior load (40). As the external load increases, the flexion of the torso increases significantly in response to the motor control mechanism to move the combined center of gravity of the body and the bag forward in order to maintain balance (21, 29, 40–43). The combination of external load and degree of torso flexion leads to increased pressure on the intervertebral discs (44–47).

This study aims to determine the change in the size of the angle of the lumbar segment of the spine in relation to the load of the average weight of SB in healthy male children (students) aged 10 to 13 years. In accordance with the reviewed literature, the hypothesis of this study is that the load of the SB significantly increases the flexion of the torso, and thus the compression of the vertebral discs. The flexion of the torso is related to the reduction of the lumbar angle formed by the examined anatomical points of the big toe, the lumbar points L3-L4 and the processus spinosus C7 (LA). The task of this research is to define the acute effect of SB load when standing and after 2-minute gait.

## Materials and methods

### Examinees

This study was a 2-factor repeated-measures design, with the participants serving as their own controls, a sample of 47 male students aged 10–13 years, mean age 12.2 ± 0.92 years, mean height 155.2 ± 8.0 cm and average body weight 45.3 ± 8.7 kg (Table 1). All students are of the same nationality, live in the same city and in the same municipality and attend the same school. Population and age were selected for this study to exclude factors of full demorphism in changes in the musculoskeletal system during the second sensitive period associated with the impact of accelerated growth and development (48). The phase of accelerated growth is a period in which posture irregularities often occur (49) and is accompanied by rapid bone growth and certain limitations of muscle elasticity, a characteristic primarily of postural muscles (50). The basic criteria for inclusion were that they always carry SB symmetrically over both shoulders, that they do not have any acute injuries and diseases and do not engage in any organized physical activity outside the school, except for physical education classes.

**Table 1:** Demographics of the subjects, male (*n* = 47).

Variables	Mean (AS)	Minimum	Maximum
Age, year	12.155 (0.9184)	9.9	13.6
Height, cm	155.162 (80.475)	139.00	180.70
Weight of subject, kg	45.336 (86.520)	30.10	70.20
Weight of school bag, kg	6.251 (1.0467)	2.3	8.1

### Study design and procedures

The experimental factor of this study represents the load in the form of SB, was defined on the basis of the weight which is conditioned by the content of SB, and in relation to the weekly school schedule (5 days a week, for 6 school lessons). The measured load of SB was 6,251 kg, that is, the load was an average 13.78% of the body weight of each subject. In addition to identifying the workload on a weekly basis, examinees were monitored from school entry to the classroom to identify how the workload was distributed, i.e., how respondents carried SB, and what time period was required to put the load down. It was found that the respondents, in 85% of cases, carried the load, SB, on both shoulders, and that on average it took about 120 s from entering the school to putting the load down (this time measure was used to assess variables in dynamic conditions).

The size of the lumbar segment (LA) angle at the L3-L4 level and its changes, caused by the additional SB load in statics and dynamics, were identified. The LA angle is defined by anatomical points: the tip of the big toe, the intervertebral space L3-L4 and the processus spinosus C7. Identification of LA size in relation to SB load in statics and dynamics was performed by photogrammetric measurement in the sport diagnostic laboratory. In the statics, the values of the angle, without and with the load SB, were identified. The values of angles in dynamics (without and with the load) were identified when walking in the phase of the middle support and the phase of initial contact of the heel, and at a speed of 4 km/h on the treadmill because it represents a comfortable, normal walking speed of children (51). In order to familiarize the subjects with the test in which angles are identified in dynamic conditions, they were exposed to a 5-minute walk (4 km/h) without load, prior to the measurement procedure (52).

The study was conducted following the ethical standards of the Declaration of Helsinki. The school and the parents have given the permission for their children's participation in this research. We have an Institutional Review Board approval document given by the Ethical Board of the Faculty of Sport and Psychology, Novi Sad, no. 423-2/2022, dated 12.05.2022.

### Variables

The variables in this study were analyzed in the sagittal plane. The flexion of the torso was analyzed on the basis of the defined lumbar angle (LA) which forms the anatomical points of the big toe, the lumbar points L3-L4 and the processus spinosus C7 in: (1) Statics (a) without SB; and (b) with SB; (2) Dynamics in the phase of middle support with the right foot (a) without SB and (b) with SB; (3) Dynamics in the phase of middle support with the left foot (a) without SB and (b) with SB; (4) Dynamics in the phase of initial contact of the heel with the right foot (a) without SB and (b) with SB and (5) Dynamics in the phase of initial contact of the heel with the left foot (a) without SB and (b) with SB. The middle phase of the support was analyzed at the moment when the weight of the body was transferred to the standing leg, and the opposite, swinging leg, was off of the ground. The initial contact was analyzed at the moment when the heel of the front foot touched the ground (48, 53).

### Tools

For the purposes of this research, two instruments were used: (1) The system (hardware and software) of the German company Contemplas was used for the estimated variables in statics. Templo software with 2D in 3 View protocol. The cameras used in the postural analysis are from the manufacturer “IDS Imaging”, model UI164xLE-C, type uEye, Firmware V1.0, USB interface, max. resolution 1280 × 1024, frequency 50 Hz. (2) For the estimated variables in dynamics, on the treadmill, Templo - Software of the German company GMBH Contemplas with software module: General Motion Analysis. Two high-speed cameras were used - Basler full HD 240 Hz. The laboratory treadmill tape is Sprintex CALLIS Z6000, with a maximum speed of 22 km per hour with a possible elevation of 15%. The lamella technology of the laboratory treadmill belt, on which we performed the test, allows a natural step, provides the best ergonomic experience of walking and running with minimal impact on the joints (51, 52). This treadmill belt has no sliding speed, it has a true zero starting speed, which is very convenient for testing and measurements. In both procedures, retroreflective markers with a diameter of 19 mm were placed on the anatomical points of the subjects for imaging. Both systems are non-invasive and completely safe for testing. The drawing and calculation of angles was done with the help of Software, marking pre-marked anatomical points with the help of retroreflective markers. On the basis of previously defined and marked anatomical points, drawing angles with tools in the software was carried out by two diagnosticians independently of each other, and under the control of a third certified photogrammetric expert who, in the case of differently plotted angles, with expert argumentation, made the final judgment on the position of the arms of the angle.

### Data analysis

The obtained results were statistically analyzed in the program SPSS V.26. package. Descriptive statistics were applied, *t*-test of paired samples was used to identify differences in LA with and without SB in statics and dynamics. Calculate the value of Cohen's *d* and the effect size (ES) correlation, r_Y_*λ*, using the *t-*test value for a between subjects *t-*test and the degrees of freedom: Cohen's *d* = 2*t* */* √ (*df*); r_Y_*λ*= √*/*[*t^2^ / (t^2^ + df)*] (54).

## Results

Table 2 shows the results of the *t*-test of paired samples which tested the difference in the size of the LA of the subjects, observed in the sagittal plane, and measured in statics. A statistically significant decrease in LA was found during SB load. The average decrease in LA was 4.953°, and the value of the effect size (ES = 0.634) indicates that the impact of the SB load is significant.

**Table 2:** Influence of SB load on LA size change in statics.

Variable	AS_1_	AS_2_	t	Df		*p*	ES
LA	167.238	162.285	8.926	46		0.000	0.634

AS_1_, arithmetic mean for LA with SB; AS_2_, arithmetic mean for LA without SB; t, t-test value; df, degrees of freedom; *p*, t-test significance; ES, effect size.

Tables 3, 4 show the results of the *t*-test of paired samples, which tested the difference in the size of LA of the subjects, observed in the sagittal plane, and measured in dynamics. In this test, LA was observed during the middle support with the right / left foot, i.e., in the phase of initial contact. For the phase of the middle support on the right foot (Table 3), a statistically significant decrease in LA was determined during SB load. The average decrease in LA was 6.295°, and the value of the effect size (ES = 0.706) indicates that the impact of the SB load is significant. The value of the change in LA at the phase of the middle support with the left foot (Table 3) indicates a statistically significant decrease in LA during SB load. The average decrease in LA was 6.664°, and the value of the effect size (ES = 0.792) indicates that the impact of the SB load is significant (Table 3).

**Table 3:** Influence of SB load on the change in the size of LA at the phase of the middle support for the right/left leg.

Variable	AS_1_	AS_2_	t	df	*p*	ES
LA right support	159.272	152.977	10.514	46	0.000	0.706
LA left support	159.743	153.079	13.243	46	0.000	0.792

AS1, arithmetic mean for LA with SB; AS2, arithmetic mean for LA without SB; t, t-test value; df, degrees of freedom; *p*, t-test significance; ES, effect size.

**Table 4:** Influence of SB load on LA size change at initial contact phase for right / left foot.

Variable	AS_1_	AS_2_	t	df	*p*	ES
LA initial contact right	142.000	134.668	12.832	46	0.000	0.782
LA initial contact left	141.617	134.511	13.150	46	0.000	0.790

AS1, arithmetic mean for LA with SB; AS2, arithmetic mean for LA without SB; t, t-test value; df, degrees of freedom; *p*, t-test significance; ES, effect size.

Table 4 shows the values for LA with and without SB and in the initial contact phase for the right/left leg. Statistically significant values can be observed for both left and right leg. The average reduction of LA at the initial contact for the right leg was 7.332°, and the value of the effect size (ES = 0.782) indicates that the impact of the SB load is significant. Also, a statistically significant change of the value in LA with the load in the phase of initial contact for the left leg is also observed. The average decrease in LA was 7.106°, and the value of the effect size (ES = 0.790).

## Discussion

In this study, the effects of short-term SB load on the lumbar segment, i.e., the change in angle in the L3-L4 level of students aged 10–13 years, in statics and dynamics, were investigated. For all examinees, the average weight of SB (6,251 kg) was defined, which is coordinated with the weekly school schedule. The load of SB at the group level was 13,78% of the average body weight of the subjects. This value can also represent a limitation of the study, because the load is not defined in relation to body mass and for each subject individually. At the group level in accordance with the defined workload of SB, it was found that there were changes in LA in statics with load, that is, the measured angle was reduced by an average of 4,953° and the value of ES (0.634) indicates that the impact of the load is significant. This result is in accordance with previous studies of the impact of SB load, which ranged from 10%–20% of the average body weight of the subjects (20–23). In the studies that treated the problem of changing the angle of the torso under load, it was found that the forward inclination of the torso in relation to the vertical line, caused by the load SB, ranges from 3.02° to 6.8° (31, 55, 56).

In our study, the phases of the middle support and the initial contact for the left and right foot when walking on a treadmill in a time frame of 110–120 s, were also analyzed. The change of LA at SB load was also identified, and the values for both tested phases ranged from 6.295° to 7.332°, while the ES value ranged from 0.706 to 0.792, which indicates that the effect of load for all four variables tested in dynamics was significant. Studies that have treated the problem of SB load (10%–20% of total body weight) in dynamic conditions and changes in the forward inclination of the of the torso in relation to the axial line, identified values ranging from 4.55° to as high as 19.80° (18, 19, 21, 28–30, 32, 33), which is also confirmed in this study. Differences in the range of angle changes in these studies can be explained by various experimental factors such as: anthropological differences in children (students) from different areas, gender, whether the dynamics were analyzed in the laboratory or open field, time spent in the dynamics after which the change in the inclination of the torso was analyzed, the weight and composition of the load filled SB (sand, weights, books, etc.) and the height at which their center of gravity was adjusted relative to the back of the subjects, etc. Therefore, the closest study to this one in relation to experimental factors is the study of Lee, Hong and Robinson on 15 boys, average age 10.36 years, with SB load of average weight of 10% of body weight, after 1 min gait on the treadmill, verified the change of the torso angle of 4.55° in relation to the torso angle without load SB (28). The results of studies in 10-year-old boys walking on a treadmill, where the average weight of SB was 15% and 20% of body weight, also identified a great influence on the change in the angle of the torso. In the same studies, when the load was 10% of the body weight of the subjects, i.e., without the SB load, there was no significant effect on the change in the angle of the torso (19, 21). The studies that have treated different weights, i.e., SB load on subjects aged 10–13 years, found that any load greater than 10% relative to average body weight has a large impact on torso inclination and LA change (18, 30).

The child's skeleton has a large amount of cartilage, significantly in the region of the spine as a prerequisite for growth. These cartilaginous regions consist of articular cartilage, epiphyses, and apophysis (24). Each of these structures is subject to different types of injury. Articular cartilage is susceptible to current stress, while the epiphyses and apophysis are more susceptible to recurrent microtrauma (57). Studies *in vivo* measuring intervertebral pressure, measured a pressure of about 0.3 MPa at the L3-L4 level (range 0.27–0.33 MPa) (37). When the torso was bent forward, an increase in intervertebral pressure of 2.5–3.6 times was found (45). The combination of intervertebral disc compression and torso flexion pre-induced by SB weight is a microtrauma that will cause an increase in intervertebral pressure (46, 47). Increased intervertebral pressure may be the cause of the accelerated process of disc degeneration (58, 59). In such a degenerated disc, the fluid level decreases, while the nucleus pulposus is not able to maintain uniform pressure on the adjacent fibrous ring and end plates (46, 47, 60). The fibrous ring consists of several nerve endings in its posterior part (61). The concentration of stress due to compression and flexion of the torso is maximal in the posterior part of the fibrous ring and irritates nerve endings that mechanically cause acute but can also be the cause of chronic lower back pain - lumbar syndrome (LS) (60, 62). LS is a major problem in the population of 40% of young people aged 9 to 18 worldwide (20, 63). The load of SB, which is greater than 10% of the total body weight (as is the case in this study), has a negative effect and contributes to the development of LS in students (20, 64). The fact that the painful condition in LS is associated with SB load was identified in 82% of students aged 11 to 14 years (65). These data are worrying because LS in youth plays an important role in the development of chronic LS in adulthood (20, 66). However, the negative impact of SB can be prevented, primarily by limiting the weight to a maximum of 10% in relation to the total body weight of the child, which can have a positive impact on the spine and thus on quality of life in later years (67). Also, it was found that the design of SB (55) and the acquisition of knowledge about its proper wearing (68, 69) can significantly mitigate its negative effects during statics and dynamics.

## Conclusions

The innovativeness of this study is reflected in the fact that for the first time in children (students), the change of LA at the level of L3-L4 with and without SB load was analyzed by photometric method in statics and dynamics. The study found that the average weight of SB was 13.78% of the average body weight of the tested sample. The results showed that the influence of SB load on LA change is great both in statics and dynamics, which is in line with previous research where SB load is greater than 10% of body weight, and, as such, causes a negative effect in terms of degenerative changes. Slightly greater changes in LA in dynamics relative to statics point to differences in the motor control strategies children use to maintain balance. Cumulatively, the microtrauma caused by SB load significantly increases the intervertebral pressure in a very critical segment, L3-L4 (which is susceptible to degenerative processes) and which can be the cause of LS. Preventive measures are needed in order to lighten SB in this population and introduce up to 10% of the child's body weight to the safe zone, as well as designing the most anatomically functional SB and educating about its proper carrying.

## Data Availability

The raw data supporting the conclusions of this article will be made available by the authors, without undue reservation.
